# Oncogenic human papillomavirus in breast cancer: molecular prevalence in a group of Congolese patients

**DOI:** 10.1099/acmi.0.000216

**Published:** 2021-03-23

**Authors:** Anicet Luc Magloire Boumba, Dimitri Malanda Mboungou Moudiongui, Sidney Frousse Christian Ngatali, Ragive Parode Takale, Donatien Moukassa, Jean Félix Peko

**Affiliations:** ^1^​ Faculté des Sciences de la Santé, Université Marien NGOUABI, Brazzaville, Republic of the Congo; ^2^​ Laboratoire d’Analyses Médicales, Hôpital Général de LOANDJILI, Pointe-Noire, Republic of the Congo; ^3^​ Laboratoire de Biologie Moléculaire HDL, Polyclinique Marie Madeleine GOMBES, Pointe-Noire, Republic of the Congo; ^4^​ Service de laboratoire d’Anatomie et cytopathologie, Centre Hospitalier et Universitaire de Brazzaville, Brazzaville, Republic of the Congo

**Keywords:** human papillomavirus DNA, human breast cancer, CHU-B, Congo

## Abstract

**Background:**

Since the work of Band *et al*. in 1990 (*Proc. Natl. Acad. Sci. USA* 87:463–467), several studies have suggested a possible link between the pathogenesis of breast cancer and viral infection. Infection with oncogenic HPV has been one of the viruses implicated in breast cancer cases worldwide.

**Objective:**

To investigate the presence of HPV DNA in archived paraffin-embedded breast cancer cases at the University Hospital of Brazzaville and to assess the association between viral HPV infections and clinicopathological features.

**Methods:**

A total of 40 formalin-fixed paraffin-embedded (FFPE) biopsies were retrospectively collected and available information was recorded. HPV detection and genotyping were performed by real-time PCR by GeneXpert technology (Cepheid, Sunnyvale, USA).

**Results:**

The mean age was 51.1±11.4 years (range 22–75 years; median was 47). Overall, HPV DNA was detected in six (15%) breast carcinoma samples. HPV-16, the most common genotype was identified in 83.7 % of all samples. HPV porting with clinicopathological features showed no significant difference (*P*>0.05). However, a statistically significant difference was observed between HPV infection and SBR grade (*P*=0.05).

**Conclusion:**

Our study described a high prevalence of HPV-HR in breast cancer cases in the Congolese woman. Future type case-control studies are necessary to better describe the potential role of HPV in the occurrence of breast cancer in Congo.

## Background

Breast cancer is the second most common cancer worldwide and the first female cancer, with 2088849 new cases diagnosed in 2018, accounting for 12.19% of all cancers. It is the leading cause of cancer death among women in developing countries with more than 324000 deaths [1]. In Africa, a total of 168690 new cases of breast cancer in women were estimated in 2018, accounting for 19.93% of all breast cancer cases [1]. In Congo it ranks second after prostate cancer with 394 new cases diagnosed in 2018 and accounts for 17.30% of all cancers. In women, it ranks first in all cancers and the second leading cause of death [1]. Today, several risk factors for breast cancer are known, such as family history, advanced age, precocious puberty, late menopause, nulliparity, and obesity. However, no factor could be directly involved in the etiopathogenesis of this cancer, except for the inheritance of certain predisposition genes, in particular the BRCA1 and BRCA2 genes. These genes are involved in 5–10% of breast cancer cases [2]. Nevertheless, certain etiological factors are more and more suspected of contributing to the development of breast cancer, among which are viral infections. In fact, about 20% of all cancers diagnosed worldwide are attributable to infectious agents, and this proportion reaches 50% in Africa [3–6]. Therefore, some viral infectious agents are considered plausible risk factors for breast cancer, including mouse mammary tumour virus (MMTV), bovine leukaemia virus (BLV), human papillomavirus (HPV) and the Epstein–Barr virus (EBV). Regarding HPV and breast cancer, it has been shown that HPV types 16 and 18 can immortalize normal breast epithelium [4, 7–9]. Since the first HPV DNA sequences were reported in breast cancer samples in 1992, there have been no studies investigating HPV DNA in Congo for women with breast cancer [7, 10]. In this work, we investigated the presence or not of HPV DNA in breast cancer biopsies. Thus, the aim of this study was to identify HPV DNA sequences and oncogenic genotypes in breast cancer paraffin-embedded biopsies obtained from Congolese women.

## Results

### Histopathological characteristics

The mean age was 51.1±11.4 years (22–75 years). The median was 47 years old. The localization of tumours according to laterality indicated that the right breast was slightly more affected (52.5%) than the left breast (45%). The histological types of non-specific type carcinoma (50%) and Grade III SBR (50%) were the most represented.

### Prevalence of HPV infection and genotypes

Table 1 shows the prevalence of HPV infection in breast cancer biopsies and the different identified high-risk genotypes. Overall, 34 (85%) were negative and six (15%) were positive to HPV-DNA, five (83.3%) were positive for HPV 16, one (16.7%) was positive for high-risk HPV other than HPV 16 or HPV 18/45. Distribution of HPV infection according to clinicopathological features is reported in Table 2. There was no statistically significant difference between HPV infection and patient age, mammary location, histological type or grade of Scarff–Bloom–Richardson (SBR) (*P*>0.05).

**Table 1.** Prevalence of HPV infection and genotypes

Characteristics	Effectives (*n*)	Frequencies (%)
**HPV infection (*n*=40**)		
HPV (+)	6	15
HPV (-)	34	85
**HPV genotypes (*n*=6)**		
HPV-16	5	83.3
HPV-X	1	16.7

HPV-X, no identified.

**Table 2.** Distribution of HPV infection according to clinicopathological features

Clinicopathological features	Total (*n*=40)	HPV infection		*p-value*
		HPV +, *n* (%)	HPV -, *n* (%)	
**Ages (years)**				
≤40	9	0 (0)	9 (100)	
41–56	16	4 (25)	12 (75)	0,27
57–71	10	2 (20)	8 (80)	
≥72	5	0 (0)	5 (100)	
**Lateralities**				
Left	21	4 (19,1)	17 (80,9)	
Right	18	2 (11,1)	16 (88,9)	0,71
Unknown	1	0 (0)	1 (100)	
**Histological Types**				
Non-specific type carcinoma	20	5 (25)	15 (75)	
Lobular carcinoma	15	0 (0)	15 (100)	
ADK little differentiated	1	0 (0)	1 (100)	0.12
ADK M differentiated	1	0 (0)	1 (100)	
ADK mucinous	2	1 (50)	1 (50)	
Medullary ADK	1	0 (0)	1 (100)	
**Histopronostic Grade (SBR)**				
Grade I	1	0 (0)	1 (100)	
Grade III	19	4 (21.1)	15 (78.9)	0.50
Grade III	20	2 (10)	18 (90)	

ADK, adenocarcinoma; SBR, Scarff–Bloom–Richardson.

## Discussion

Breast cancer is the leading cause of cancer death in women worldwide, with an incidence that is increasing every year [1, 11]. Several breast cancer risk factors were identified: age, family and genetic history. However, 50–80% of breast cancer remains without apparent cause [12]. The role of viral infections in the genesis of certain cancers has been formally established. Viruses contribute to about 20% of cancers [6, 13, 14]. The link between HPV and cervical cancer is well established. However, the involvement of this virus as an etiological risk factor for breast cancer remains controversial [13, 15]. Band *et al*. (1990) were the first to postulate that HPV might be involved in breast cancer [9].

To our knowledge, few data are available on the prevalence of HPV in breast cancer cases in Africa. We report here the first Congolese descriptive study evaluating the presence of HPV-DNA in paraffin-embedded breast cancer biopsies using the Cepheid Xpert-HPV test.

The Cepheid Xpert-HPV assay is a new method for HPV testing using the liquid phase cytology samples. Several studies have also evaluated Xpert-HPV assay as a powerful method for amplifying oncogenic HPV in literature using cervical smear samples [16–18]. However, few authors have used Xpert HPV assay to detect oncogenic HPV in formalin-ﬁxed parafﬁn-embedded samples. Indeed, the Xpert HPV is a qualitative test which detects the 14 HR-HPV types based on a real-time PCR. Guerendiain *et al.* (2016), Virtanen *et al.* (2017) and Donà *et al.* (2017) were the first authors to use GeneXpert technology for amplification of HPV in formalin-ﬁxed parafﬁn-embedded samples. The results obtained by these studies have shown a perfect concordance with other conventional techniques, which shows that the Xpert HPV system provides a convenient molecular method for testing FFPE samples [19–21].

In our study, the overall prevalence of high-risk HPV infection was 15%. Our results corroborate the majority of studies that investigate the presence of viral HPV-DNA in cases of breast cancer worldwide [12, 22, 23]. However the prevalence observed in our study was low compared to other studies conducted worldwide [22, 24, 25]. However, the relative size of our sample could not allow us a good comparison of prevalence with the other studies and the descriptive character of our study rightly limits its scope. However, this study provides evidence of the presence of HPV DNA in breast cancer biopsies in the Congolese population. In general, HPV DNA in breast cancer samples has been detected in several African countries with different prevalences especially in Algeria, Morocco and Rwanda [22, 26, 27]. In fact, Band *et al.* (1990) was the first author to postulate that HPV could be involved in breast cancer and Di Lonardo *et al.* (1992) showed by molecular amplification the presence of HPV-16 in 29.4 % of breast cancer samples, corroborating a potential relationship between HPV and breast cancer [9, 10]. The HPV 16 genotype was the most common in our study. These findings are consistent with those of other authors in the literature [22, 24, 28]. It is known that the HPV 16 genotype is the most oncogenic and responsible for more than half of cervical cancer cases [13, 29, 30]. In our study, only one case of HPV could be genotyped. This type is certainly a low-risk HPV genotype, which is not taken into consideration by the Xpert HPV system. This Xpert HPV system can genotype all oncogenic HPV types by formally identifying the HPV 16 on the one hand, the HPV18/45 on the other hand and other high-risk HPVs grouped together.

In fact, it should be noted that the Xpert HPV technique is ideal for analysing and identifying papillomaviruses in paraffinic biopsies. This system also has the advantage of partial typing of high-risk HPV 16, 18/45 genotypes responsible for more than 90% of cervical cancer cases worldwide.

Moreover, in the case of breast cancer, although our study has been able to show the presence of HPV DNA in our series, many studies have not identified HPV in cases of breast cancer [31, 32]

The main limitation in this work is the small sample size. However, our preliminary study had the privilege of showing the presence of HPV-HR DNA in certain biopsies of breast cancer in the Congolese population. This opens the way to more important investigations for the verification of this research component.

## Conclusion

Human papillomavirus was found in 15% of breast cancer cases in our study. It is an informative study because it shows the molecular signature of high-risk HPV in breast cancer cases in our country. It also shows the predominance of HPV16 in all HPV-positive cases although no significant association with histological types was observed. A larger study is needed to confirm this hypothesis and to consider infectious causes in the management of breast cancer in Congo.

## Methods

### Study samples

A descriptive and cross-sectional study was conducted on biopsies of breast cancer included in paraffin blocks between 2014 and 2017. The data collection was retrospective by analysis of the registers of the results relating to cases of breast cancer.

**Fig. 1.**
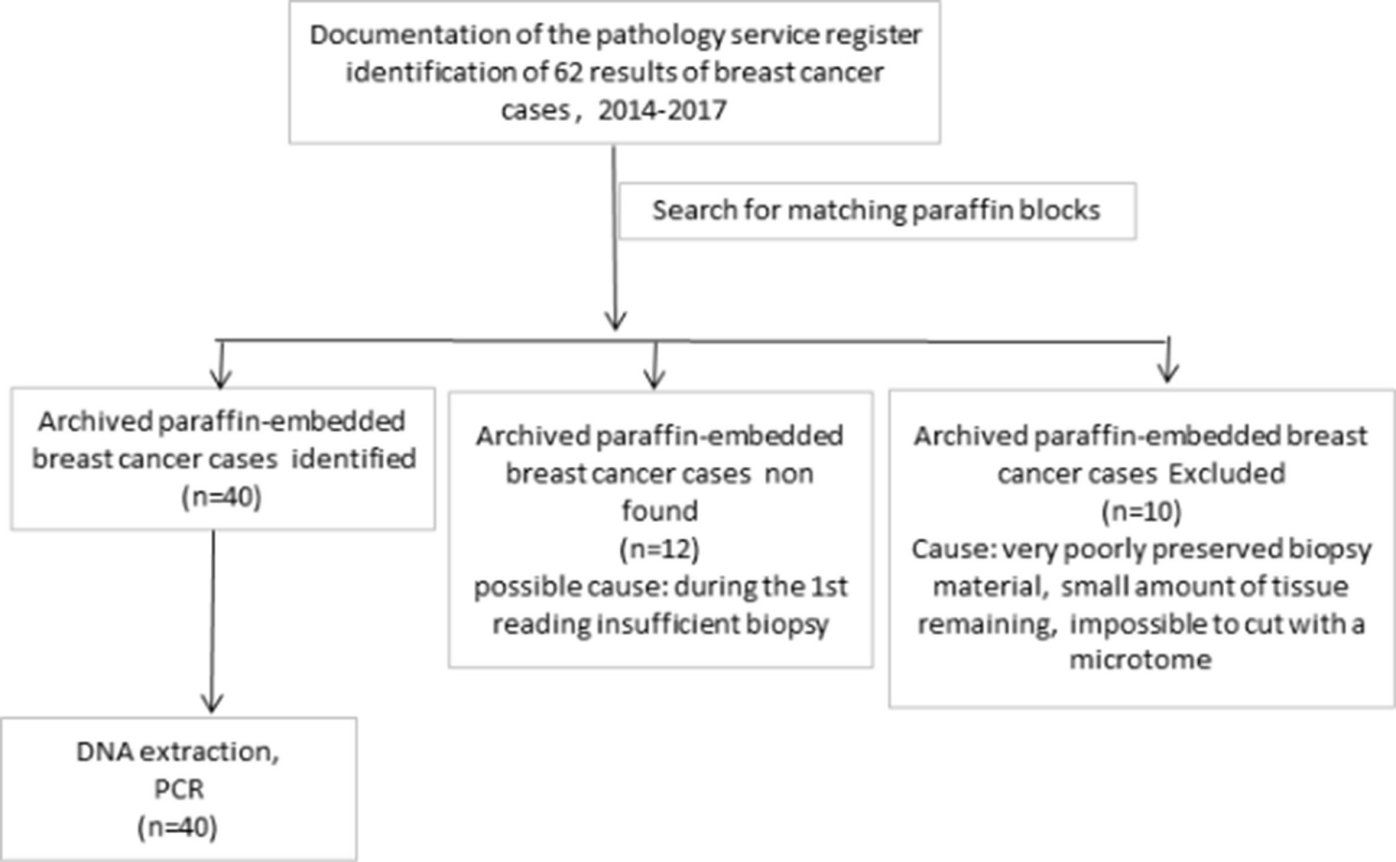
Paraffin block selection flowchart.

In fact, 62 files meeting the major selection criteria ‘breast cancer’ were identified in the service’s registers. Twelve blocks of paraffin could not be found in the archives and 10 blocks were excluded for poor conservation of the material which had led to their unexploitation (Fig. 1). The study therefore focused on 40 archived paraffin-embedded breast cancer cases, diagnosed between 2014 and 2017, extracted from the archives of the pathology laboratory of the University Hospital Centre of Brazzaville. Information on the clinicopathological characteristics (age, sex, tumour localization, histological type and Scarff–Bloom–Richardson grade) of the patients was used in patient records. No data could be found on the different phenotypic characteristics such as the expression of hormone receptors (oestrogen receptors (ER) or progesterone (PR)) and overexpression of the human epidermal growth factor receptor 2 (HER2 new). The biopsies or surgical specimens used in this study were fixed in 10% neutral buffered formalin before paraffin embedding. In fact, 4 µm sections were cut microtomically for each of the 40 paraffin blocks of the breast cancer tissues. Tissue ribbons were placed on slides for hematoxylin and eosin (H and E) staining. The slides were then deparaffinized in three toluene baths for 5 min each, and then rehydrated with a series of alcohol baths at decreasing degrees (100, 95 and 80%) for 3 min each. The sections were then stained with hematoxylin for 5 min, rinsed gently in water for 10 min and stained with eosin for 1 min. The slides were subsequently dehydrated with a growing series of alcohol (80, 95 and 100% ethanol). After immersion for 5 min in a toluene bath, they were mounted with covered coverslips using a mounting medium (Eukkit).

HPV detection and genotyping were performed using the Cepheid GeneXpert Instrument Systems (Cepheid, Sunnyvale, USA) which is a real-time PCR in the Molecular Biology Laboratory of the Marie Madeleine Foundation in Pointe-Noire.

### DNA extraction

#### Pretreatment of the samples

It was performed in two steps, dewaxed with xylene and enzymatic digestion with proteinase K. Sections of 5 µm were obtained using a microtome and the dewaxing was performed according to the xylene-ethanol method for all paraffin-embedded biopsies. The dewaxed tissue was washed with PBS solution. Then we performed enzymatic digestion with proteinase k concentrate at 2 mg ml^−1^

#### Extraction of the DNA

The ‘RNA / DNA purification kit’ (NORGEN) was used for the extraction of DNA. Once the tissue was digested, we added 800 µl of ethanol at 90 °C (Cooper) to each tube containing the samples. The mixture was homogenized and extracted according to the manufacturer’s instructions. The obtained DNA extracts were measured with a Qubit 3.0 fluorometer to evaluate the concentration of the DNA.

### HPV detection

#### Assay description

The Xpert HPV assay is an automated test for qualitative detection and differentiation of HPV DNA. The assay is performed on Cepheid GeneXpert Instrument Systems. GeneXpert Instrument Systems automate and integrate sample processing, cell lysis, purification, nucleic acid amplification, and detection of the target sequences in clinical samples by using real-time PCR. The systems consist of an instrument, personal computer, and preloaded software for running tests and viewing the results. The systems require the use of single-use disposable GeneXpert cartridges that hold the PCR reagents, house the sample, and carry out the PCR processing. Because the cartridges are self-contained, cross contamination between samples is minimized. For a full description of the systems, refer to the appropriate GeneXpert Dx System Operator Manual or the GeneXpert Infinity System Operator Manual. The Xpert HPV Assay includes reagents for the detection of high risk HPV.

The Xpert HPV is a qualitative *in vitro* test for the detection of the E6/E7 region (targets a sequence of 80–150 bp) of the viral DNA genome from high-risk human papillomavirus (HPV) in patient samples. The assay performs multiplex amplification of the target DNA rt-PCR of 14 high-risk HPV genotypes in a single run. Xpert HPV specifically identifies HPV 16 and HPV 18/45 genotypes in two separate detection channels and reports 11 other high-risk genotypes (31, 33, 35, 39, 51, 52, 56, 58, 59, 66 and 68) in a pooled result.

Following the manufacturer’s instructions for collecting cervical specimens, a Sample Adequacy Control (SAC) and a Probe Check Control (PCC) are also included in the cartridge. SAC reagents detect the presence of a single copy human gene and monitor whether the specimen contains adequate numbers of human cells to carry out a qualitative assessment of HPV status. The PCC verifies reagent rehydration, PCR tube filling in the cartridge, probe integrity, and dye stability. The six colour channels contain primers and probes for the detection of specific genotypes or pooled results as follows: ‘SAC; Primary’ for the Sample Adequacy Control, ‘HPV 16; Primary’ for HPV 16, ‘HPV 18_45; Primary’ for the HPV 18/45 pooled result, ‘P3; Primary’ for the pooled result of any of HPV types 31, 33, 35 52, or 58, ‘P4: Primary’ for the pooled result of either of HPV types 51 or 59, and ‘P5; Primary’ for the pooled result of any of HPV types 39, 56, 66 or 68.

#### Statistical analysis

All statistical analyses were performed using the Epi-info V7.2 statistical package. The Chi-square test was used for the comparison of the variables with HPV infection. The *p*-values (*P*≤0.05) were considered statistically significant.
